# Comparative Analysis of Ethylene/Diene Copolymerization and Ethylene/Propylene/Diene Terpolymerization Using Ansa-Zirconocene Catalyst with Alkylaluminum/Borate Activator: The Effect of Conjugated and Nonconjugated Dienes on Catalytic Behavior and Polymer Microstructure

**DOI:** 10.3390/molecules26072037

**Published:** 2021-04-02

**Authors:** Amjad Ali, Muhammad Khurram Tufail, Muhammad Imran Jamil, Waleed Yaseen, Nafees Iqbal, Munir Hussain, Asad Ali, Tariq Aziz, Zhiqiang Fan, Li Guo

**Affiliations:** 1 Research School of Polymeric Materials Science & Engineering, Jiangsu University, Zhenjiang 212013, China; 11629042@zju.edu.cn (A.A.); waleed8205@yahoo.com (W.Y.); nafeesiqbal11@gmail.com (N.I.); 2 School of Chemistry and Biological Engineering, Beijing Institute of Technology, Beijing 100081, China; Khurram.ch91@bit.edu.cn; 3 MOE Key Laboratory of Macromolecular Synthesis and Functionalization, Department of Polymer Science and Engineering, Zhejiang University, Hangzhou 310027, China; jamil@zju.edu.cn (M.I.J.); munir88@zju.edu.cn (M.H.); Tariq_mehsud@yahoo.com (T.A.); 4 National Research Center of Pumps, Jiangsu University, Zhenjiang 212013, China; asadam969@gmail.com

**Keywords:** metallocene, borate, ethylene, diene, conjugated, nonconjugated, polymerization

## Abstract

The copolymerization of ethylene‒diene conjugates (butadiene (BD), isoprene (IP) and nonconjugates (5-ethylidene-2-norbornene (ENB), vinyl norbornene VNB, 4-vinylcyclohexene (VCH) and 1, 4-hexadiene (HD)), and terpolymerization of ethylene-propylene-diene conjugates (BD, IP) and nonconjugates (ENB, VNB, VCH and HD) using two traditional catalysts of C_2_-symmetric metallocene—silylene-bridged rac-Me_2_Si(2-Me-4-Ph-Ind)_2_ZrCl_2_ (complex A) and ethylene-bridged rac-Et(Ind)_2_ZrCl_2_ (complex B)—with a [Ph_3_C][B(C_6_F_5_)_4_] borate/TIBA co-catalyst, were intensively studied. Compared to that in the copolymerization of ethylene diene, the catalytic activity was more significant in E/P/diene terpolymerization. We obtained a maximum yield of both metallocene catalysts with conjugated diene between 3.00 × 10^6^ g/mol_Mt_·h and 5.00 × 10^6^ g/mol_Mt_·h. ENB had the highest deactivation impact on complex A, and HD had the most substantial deactivation effect on complex B. A ^1^H NMR study suggests that dienes were incorporated into the co/ter polymers’ backbone through regioselectivity. ENB and VNB, inserted by the edo double bond, left the ethylidene double bond intact, so VCH had an exo double bond. Complex A’s methyl and phenyl groups rendered it structurally stable and exhibited a dihedral angle greater than that of complex B, resulting in 1, 2 isoprene insertion higher than 1, 4 isoprene that is usually incapable of polymerization coordination. High efficiency in terms of co- and ter- monomer incorporation with higher molecular weight was found for complex 1. The rate of incorporation of ethylene and propylene in the terpolymer backbone structure may also be altered by the conjugated and nonconjugated dienes. ^13^C-NMR, ^1^H-NMR, and GPC techniques were used to characterize the polymers obtained.

## 1. Introduction

The design and production of well-defined, single-site catalysts from coordination compounds has generated new opportunities for academic and industrial researchers for the synthesis of polyolefin with the precise control of the chain structure and physical properties [1,2,3,4]. The co-/terpolymers of linear conjugated and cyclic nonconjugated dienes with ethylene or propylene are expected to show numerous unique properties, such as excellent transparency, high tear and tensile strength, low environmental stress cracking, good resistance to water/heat/chemicals, and thermoplasticity ranging from elastomer to highly crystalline solids [5,6,7,8]. These properties depend on the polymer microstructure and dienes content, i.e., 2–8 wt% of diene is the basic demand of industrial applications [9,10]. Ethylene propylene diene monomer (EPDM) elastomeric materials are a promising class of thermoplastics with versatile applications widely used in outdoor applications like the roofing of buildings, sealing of automotive windows/doors, and drinking water due to chloramine and chlorine resistance. These properties depend on several parameters, such as co/ter monomer type, composition, and chain distribution. In addition, Wang and co-workers determined that the long-chain branching in metallocene-catalyzed olefins polymerizations was the determining factor in controlling the rheological properties of the EPDM [9,10,11]. An ethylene-propylene copolymer produced with a heterogeneous Ziegler-Natta catalyst was investigated by Dong et al. [12]. They suggest that the arrangement of polymer fractions has become blockier owing to a rise in ethylene content. The incorporation of the third monomer, such as dienes, was found to improve the ethylene/propylene elastomers’ properties. The ones most commonly used as co/ter monomers in industrial EPDM are the cyclic nonconjugated dienes (ENB, VNB and DCPD), exhibiting a strained double bond reactive in metallocene-catalyzed olefin polymerizations. Moreover, unreactive double bonds (1, 2 disubstituted or trisubstituted) of nonconjugated dienes can be utilized for different postpolymerization techniques [12,13,14,15,16]. However, despite these advantages, the use of linear conjugated dienes (IP and BD) can also enhance EPDM production with improved properties by regioselectivity insertion into the co/ter polymers backbone. The introduction of conjugated dienes not only reduces the cost of manufacturing, but also incorporates unsaturated bonds into the ethylene backbone, giving the resulting copolymers some interesting properties (high cross-linking efficiency, rubber compatibility), which are possibly beneficial in resolving the compatibility problem during its covulcanization with conjugated dienes rubber. In addition, by postfunctionalizing the double bonds in the backbone of EPDM, different polar functional groups can be inserted into the backbone with ease. However, reports of copolymerization of ethylene with conjugated diene are rare. The vanadium- (VOCl_3_, VCl_4_, VO(OR)_3_ and V(acac)_3_) and titanium-based heterogeneous Zeigler‒Natta catalysts have been used in the copolymerization of ethylene with conjugated diene. Unfortunately, the copolymerization activity was significantly reduced due to the stronger coordination efficiency of the conjugated diene than ethylene [17,18,19,20,21]. It is difficult to use vanadium (V) since discoloration, ageing, and toxicity are induced by the polymer’s residual V content over 5–10 ppm. Additionally, in the presence of dienes, these vanadium-based catalysts dramatically lose potency. Furthermore, due to crosslinking and side reactions during terpolymerization, the polymer’s diene content is reduced [22]. More uses in industrial polyolefin processing have been discovered recently through single-site catalysts. Single-site catalysts’ ability to copolymerize ethylene with a-olefins in a homogeneous manner is a well-known distinguishing function. Compared to the heterogeneous Zigler–Natta (Z–N) catalyst, single-site metallocene has a benefit in the manufacture of EPDM, both in preventing the usage of toxic V-based and utilizing the well-known high activity of metallocene [23,24]. These catalysts can allow for random monomer distributions and have substantial control over the distribution of molecular weight. Many researchers have thoroughly studied the metallocene catalysts and recommend that these catalysts have the potential to produce higher polymerization activity and a higher incorporation rate of comonomer and as well as termonomer in the polymer backbone without any side reactions [25,26]. A suitable choice of catalysts, a cocatalyst and activator, and types of diene could modulate EPDM synthesis. In addition, metallocene Cp_2_ ZrCl_2_/MAO, *rac*-Me_2_Si(2-Me-4-Ph-Ind)_2_ZrCl_2_/MAO, and Et(IndH_4_)_2_ZrCl_2_/MAO presented higher productivity in co/ter-conjugated and nonconjugated dienes polymers [27,28]. According to the literature, the copolymerization of ethylene with conjugated linear dienes is challenging because butadiene and isoprene show very different reactivity appearances for a homogeneous metallocene and heterogeneous Z–N catalyst and, in some cases, conjugated dienes can act as toxic substances for the polymeric catalysts. To date, several research articles have reported on this topic, but most of them have focused on E-BD’s copolymerization [29,30], while the copolymerization of ethylene with isoprene has received little attention. However, the precise control of the E-IP copolymer microstructure and IP content has continued to be a challenge for researchers. Isoprene is a very interesting and more attractive diene, and is commercially readily available at a low price. Moreover, the methyl group substituents could force it to react in unusual ways, leading to a new polymeric chain structure. Isoprene, cis1, 4 insertion is a key component of natural rubber and produces an outstanding elastomer, while trans-1, 4, and iso-3, 4 isoprene insertion are crystalline forms. However, 1, 2 insertion of isoprene is generally rarely available in coordination polymerization for steric reasons [5,31,32].

As we studied before, the rac-Me_2_Si (2-Me-4-Ph-Ind)_2_ZrCl_2_ (complex A) methyl and phenyl groups render it structurally more stable and contribute a greater dihedral angle and polypropylene with higher activity and molecular weight that is more stereospecific, as with isotacticity polypropylene (isotactic pentad fraction [mmmm]) and its copolymerization with ethylene [31]. Similarly, Et(Ind)_2_ZrCl_2_ (complex B) is one of the simplest C_2_-symmetric metallocene catalysts, comprehensively studied and well known to produce ethylene with ENB copolymers with 5–10 mol% [32,33,34]. Motivated by these results, the copolymerization of ethylene with diene, conjugated (IP, BD) and nonconjugated (HD, VCH, ENB, VNB), and terpolymerization of ethylene, propylene, and diene, conjugated (IP, BD) and nonconjugated (HD, VCH, ENB, VNB), was carried out, catalyzed by complex A and complex B with TIBA/borate cocatalyst combination. The evident influence of conjugated and nonconjugated dienes’ structure on polymerization productivity, behaviors, and polymer structure has been noted. This study focuses on the effects of the amount and type of dienes in E-diene copolymers and E-P-dienes’ terpolymerization. With the objective of synthesizing ethylene-diene and ethylene-propylene-diene terpolymers with a more controllable chain structure and molecular weight distribution, the use of an atom or groups of atoms in metallocene bridges and in the ligand structure directly affect dienes’ incorporations. NMR, GPS and DSC characterized the polymers obtained. The determination of co-/terpolymer microstructure, comonomer and termonomer content, chain end groups, thermal properties, and molar mass led to insight into the factors that affect the co/ter monomer insertion and chain transfer reaction.

## 2. Experiments

### 2.1. Materials

The polymerization catalyst, *ra*c-Me_2_Si (2-Me-4-Ph-Ind)_2_ZrCl_2_ (complex A), was provided by the Shanghai Research Institute of Chemical Industry (Shanghai, China); *ra*c-Et(Ind)_2_ZrCl_2_ (complex B) (less than 3% of CH_2_Cl_2_) was purchased from Sigma-Aldrich, Shanghai, China. Butadiene (BD) (Sinochem Lantain Zhejiang Research Institute of Chemical Industry Co., Ltd., Hangzhou, China) and donated borate activator (Ph_3_C)B(C_6_F_5_)_4_ were used according to guidelines from the published literature [33,34]. TIBA (99%) was obtained from Albemarle Co. Beijing, China, and diluted using Schlenk line techniques into n-heptane (2M solution) and stored in a desiccator. Ethylene (polymerization grade, 99.9% purity) and propylene (polymerization grade, 99.9% purity) were bought from Zhejiang Minxing Gas Co. (Hangzhou, China) and further purified by passing through columns containing a deoxygenizing agent and 4 Å molecular sieves in a gas purification technique (Dalian Samat Chemicals Co., Ltd., China). Toluene (HPLC grade) was purchased from Jiangsu Yonghua Fine Chemical Co., Ltd. (China), further purified by refluxing over sodium benzophenone, and collected by the distillation method under dry nitrogen before polymerization tests. 5-Ethyli, dene-2-norbornene (ENB) (99 wt% solution in heptane, containing 80% of cis-isomer) was bought from Arcos Organics, Shanghai, China. 1, 4-hexadiene (HD) (99%), and 4-Vinylcyclohexene (VCH) (98%) were bought from Sigma-Aldrich, Shanghai, China. Butadiene (BD, polymerization grade) was purchased from Zhejiang Gas mixing Co. (Hangzhou, China) and further purified by passing through a column of molecular sieve, neutral alumina, and potassium hydroxide. Isoprene (97%), bought from Energy Chemical Co. (Shanghai, China), was refluxed over CaH_2_ and then distilled and stored under nitrogen. All dienes were dehydrated by molecular sieves, collected followed by a distillation method, and stored in a nitrogen atmosphere.

### 2.2. Polymerization

Using a dry glove box and Schlenk line techniques, both polymerization reactions were carried out in a dry nitrogen environment. Every day, the dry box was purged with nitrogen for 5 min before the polymerization experiments. In a 90 °C glassware oven, the 150-mL glass reactor with a stirrer was baked, and the installed glass reactor was evacuated from the vacuum line and refilled with dry nitrogen. The flask was first filled with about 50 mL toluene at 50 °C and then saturated with gaseous ethylene or ethylene/propylene (80/20) of 0.1 MPa. In toluene solvents, borate and metallocene were prepared and used for a series of five experiments. Anhydrous diene (co/ter monomer) was added to the reactor, followed by TIBA (2 mol/L solutions in n-heptane), and toluene solutions of the borate and the metallocene. Ethylene or ethylene‒propylene (80/20) with a pressure of 0.1 MPa was continuously supplied to the reactor during the experiment. After the planned time, the polymerization reaction was terminated by adding acidic ethanol (containing about 2% HCl) to decompose the metallocene and borate. The polymers were precipitated, cleaned, and dried for 24 h at 45 °C in a vacuum oven.

### 2.3. Characterization

#### 2.3.1. Spectroscopic Analysis (^13^C NMR ^1^HNMR)

Using a Varian Mercury instrument (MOE Key Laboratory of Macromolecular Synthesis and Functionalization, Department of Polymer Science and Engineering, Zhejiang University, Hangzhou, China). Plus 300 spectrometers running at 75 MHz in pulse Fourier transform mode, the ^13^C NMR spectra of the ethylene-propylene-diene conjugated and conjugated terpolymer were carried out at high temperatures. The monomers content (E and P) in the terpolymer was determined at 120 °C, using hexamethyldisiloxane as an internal chemical shift reference. The solvent was o-Dichlorobenzene-d4 and the solution had a concentration of 10% by weight. The pulse delay was fixed to 3 s, and the period for the acquisition was 0.8 s. The pulse angle was set to 90°; 8000 Hz was the spectral width. For integration, inverse gated decoupling was developed. Generally, 4000 scans were recorded [35]. 

The content of co/ter monomer dienes, e.g., ENB, VNB, HD, VCH, BD and IP, was analyzed by the ^1^H NMR spectrum, using the signals of inserted co/ter (diene) units in co/terpolymers. ^1^H NMR spectra were recorded using the same instrument. The ^1^H NMR spectra of the ethylene-propylene-diene conjugated and conjugated terpolymer were carried out at 120 °C using the same instruments. The solvent was o-Dichlorobenzene-d4 and the solution had a concentration of 4‒5% by weight. The period for the acquisition was 1.998 s and the time for pulse decay was 3.00 s. For each study, an average of 250 scans is taken [35].

#### 2.3.2. Gel-Permeation Chromatography (GPC)

GPC was used to evaluate the Mw and MWD of purified co/terpolymer samples using a PL 220 GPC instrument (Polymer Science Laboratories, Hangzhou, China) fitted with three PL-gel 10 μm MIXED-B columns with the eluent 1,2,4-trichlorobenzene at 150 °C with a flow rate of 1.0 mL/min. The decision to use a traditional calibration system was made based on the narrow polystyrene requirements.

## 3. Results and Discussion

**Figure 1:**
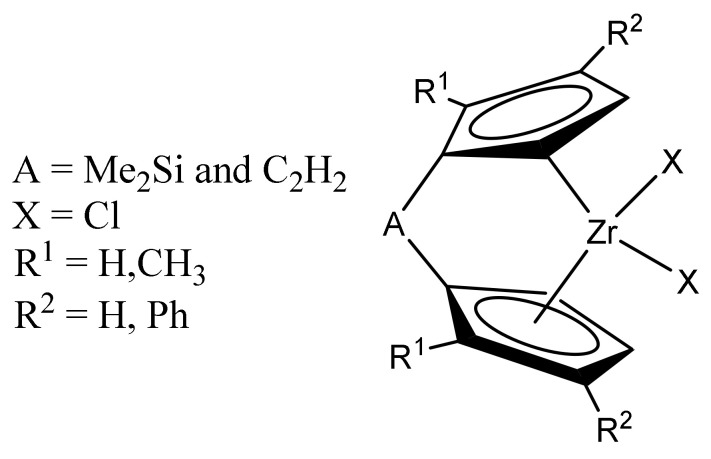
Structure of the metallocene catalyst.

Two representative ansa-metallocenes (Figure 1) were selected as the two polymeric catalyst precursors (complex A and complex B), leading to poly (ethylene-diene) and poly(ethylene/propylene-diene) with different chain structures.

### 3.1. Ethylene, Propylene, and Diene Monomer Copolymerization

We performed a comparative analysis of metallocene-based ethylene/conjugated and ethylene/nonconjugated dienes’ copolymerization under the same reaction conditions. However, in most previous studies, the homogeneous catalyst precursor was co-catalyzed with methylaluminoxane (MAO) or modified methylaluminoxane (MMAO) [36,37]. At this time, the TIBA cocatalyst was used along with a borate activator for the alkylation of homogeneous catalyst precursors, while TIBA was also an impurity scavenger. Some of the borates are very active cocatalysts, e.g., B(C_6_F_5_)_3_, [HNRR′_2_]^+^[B(C_6_F_5_)_4_]^−^, and [Ph_3_C]^+^[B(C_6_F_5_)_4_]^−^ and were used for olefin polymerization and mechanistic studies [36,38]. 

Initially, a series of ethylene-diene (conjugated and nonconjugated) copolymerizations were performed under the same reaction conditions using a ansa-metallocene/alkylaluminum/borate catalyst system; the results are summarized in Table 1 and Table 2. The number of dienes were set to 0.06 and 0.12 mol/L, and the molar ratios of Zr/Al and Zr/borate, and the other polymer reaction conditions were kept constant. The selected polymerization reaction conditions were more suitable for the catalyst activities and a diene conversion, around 5–10 mol%.

Both catalysts showed C_2_ symmetry, but different ligand structures, which means that they produce E-diene copolymers and E/P-diene terpolymers with various activity and chain microstructures depending on the electronic impact and size of ligands attached to the Zr metal. According to the literature, complex B is one of the simplest C_2_-symmetric metallocene catalysts, comprehensively studied and well known to produce ethylene with cyclic diene copolymers with 10‒15 mol% [7,39].

**Figure 2:**
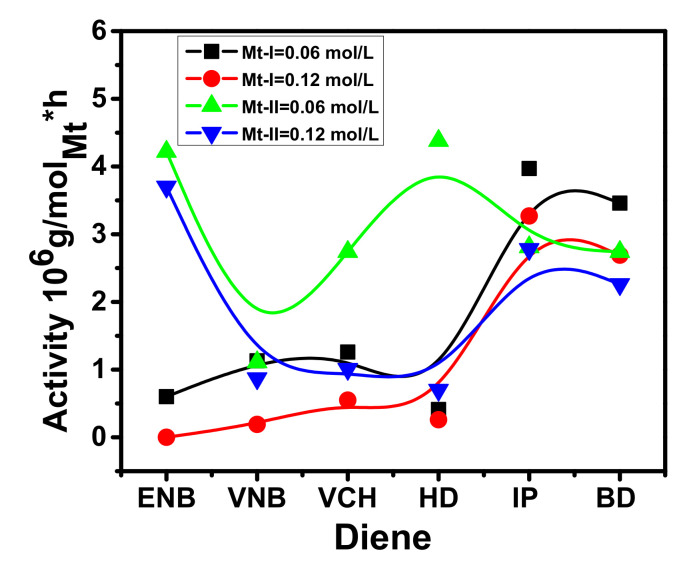
Activity of ethylene/diene (conjugated and nonconjugated) copolymerization with complex A and complex B at 0.06 mol/L and 0.12 mol/L.

Conjugated and nonconjugated dienes were copolymerized with E at 0.06 and 0.12 mol/L concentrations at the same reaction temperature. The current study found that a small number of conjugated and/or nonconjugated dienes being introduced could affect the ethylene homo and E/P copolymerization behaviors with a metallocene/borate/TIBA catalyst system. Malmberg and co-workers have reported that a large number of nonconjugated dienes may result in decreased polyolefin activity [40]. However, significant quantities of conjugated or nonconjugated dienes (5–10%) were theoretically appropriate for synthesizing the EPDM. Only a limited number of dienes have been applied to preserve the high degree of activity in the copolymerization of E/dienes. The co-polymerization activity of E/conjugated and E/nonconjugated dienes for both catalysts (complex A and complex B) are shown in Figure 2.

**Figure 3:**
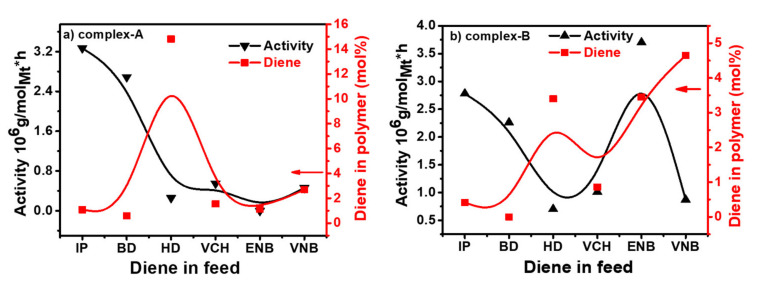
Variation of activity profile and diene content in ethylene/diene in copolymer with (**a**) complex A and (**b**) complex B.

For both catalysts, the polymer yields, molecular weight, molecular weight distribution, and diene content are listed in Table 1 and Table 2. The behavior profiles at 0.12 mol/L for different forms of conjugated and nonconjugated dienes were constructed. Figure 3 shows both catalysts to concentrate on the effect of diene content involving polymerization activity.

It was noted that the addition of conjugated dienes resulted in a slight increase in the catalytic activity, regardless of the E/conjugated diene ratios. However, reduced catalytic activity was more prominent with nonconjugated dienes, possibly because of lower E polymerization in nonconjugated dienes under the specified reaction conditions [41]. Considering that the E/conjugated diene copolymerization activity was higher than that of polyethylene (PE) because of the comonomer effect, a similar phenomenon was investigated in E/P copolymerization by Waymouth et al. and Fan et al. [10,26]. They reported that the increased rate could not be explained by the higher rate of E monomer insertion but was possibly the result of E activation of the dormant catalyst sites. Several theories have been put forward to explain this phenomenon, including the trigger mechanism studied by Naga et al. [42] and developed diffusion rates due to the active center’s solubilization through the insertion of comonomers reported by Koivumaki et al. [42]. However, it was proposed, based on the analysis, that conjugated diene would prevent ethylene insertion to the active site of the catalyst, resulting in reduced activity, so that adding propylene with a high activity ratio could improve the situation. However, it was noted that, when different forms of diene were applied to the system, as opposed to traditional ethylene‒propylene copolymerization, the activity decreased significantly [13,37]. Among the types of diene used for complex A, it should be noticed that the insertion of IP resulted in the highest polymerization activity. By contrast, adding ENB resulted in the lowest activity, presented the most substantial deactivation influence, and decreased the catalytic activity to 0 when the ENB concentration was higher than 0.6 mol/L. The deactivation magnitude caused by HD and VCH was also stronger in complex A than in complex B. Overall, complex A’s copolymerization activity was more sensitive to the nonconjugated diene concentration than complex B. The effects of BD, IP, and ENB on both catalysts were sharply different. While BD, IP and VNB had the lowest deactivation impact on complex A, they caused almost complete deactivation of complex B at a very low concentration. The activities of E/nonconjugated diene copolymerization with complex B cocatalyzed by a TIBA/borate system can be equated with those of an aluminoxane (MAO) activated complex B. According to the literature, the activity of E/VCH copolymerization with complex B/MAO was higher at 60 °C than at 30 °C and 40 °C [39]. Lower sensitivity of the system was seen at 30 °C and 40 °C. On the other hand, E/VNB copolymers with significant VNB content were fully soluble in toluene at 50 °C. The activity of E/VNB copolymerization with complex B/MAO was 4.9 kg of copolymer/g of Zr (VNB 0.2 M polymerization temperature = 35 °C, ethylene pressure = 1 atm and solvent toluene = 25 mL) [17]. Complex A has higher E/conjugated diene copolymerization activity than complex B and shows copolymers with higher conjugated diene (IP, BD) content (see Figure 2). In contrast, complex B shows higher ethylene/nonconjugated diene copolymerization activity and content than complex A. According to the literature, the addition of dienes in ethylene polymerization with complex B/MAO and complex B/MMAO caused a marked decrease in catalytic activity [43]. However, when I.P and B.D were added to complex-A, and ENB inject to complex B, the feed amount was 0.06 mol/L, led to improvements in polymerization activities (Table 1 and Table 2).

The impact of the amount of diene on the metallocene activity in the copolymerization of ethylene with ENB, IP and BD was also noted. The catalytic activity is higher than the homopolymerization of ethylene when the amount of diene is 0.06 mol/L, and decreases with increasing diene in the system. Similar phenomena were observed for the ethylene‒propylene copolymerization. Numerous reasons have been given in the literature to explain the increase in catalytic activity with low comonomer concentrations, such as the ethylene homopolymer’s low solubility compared to the ethylene copolymer, which helps with monomer diffusion to the active center [26,44]. Moreover, the comonomer plays a role in the activation of new catalyst active sites and increases the E insertion rate. Subsequently, at a particular comonomer concentration, the reaction rate decreases, possibly because of the lower insertion rate of diene (ENB, IP and BD) in contrast to E. In other words, because of steric effects, the insertion of comonomers is slower when the active species is complex A than when it is complex B. According to the literature, for homogeneous metallocene catalysts, a decline in catalytic activity was noticed since the monomer’s insertion was slow after the insertion of diene units. This difficulty can be overcome by introducing an atom or group of atoms (bridged) among the two ligands. A high insertion rate and efficient activity was obtained with catalysts *rac*-Me_2_Si (2-Me-4-Ph-Ind)_2_ZrCl_2_ and (*rac*-Et(Ind)_2_ZrCl_2_ [36].

### 3.2. Chain Structure of Copolymers

**Figure 4:**
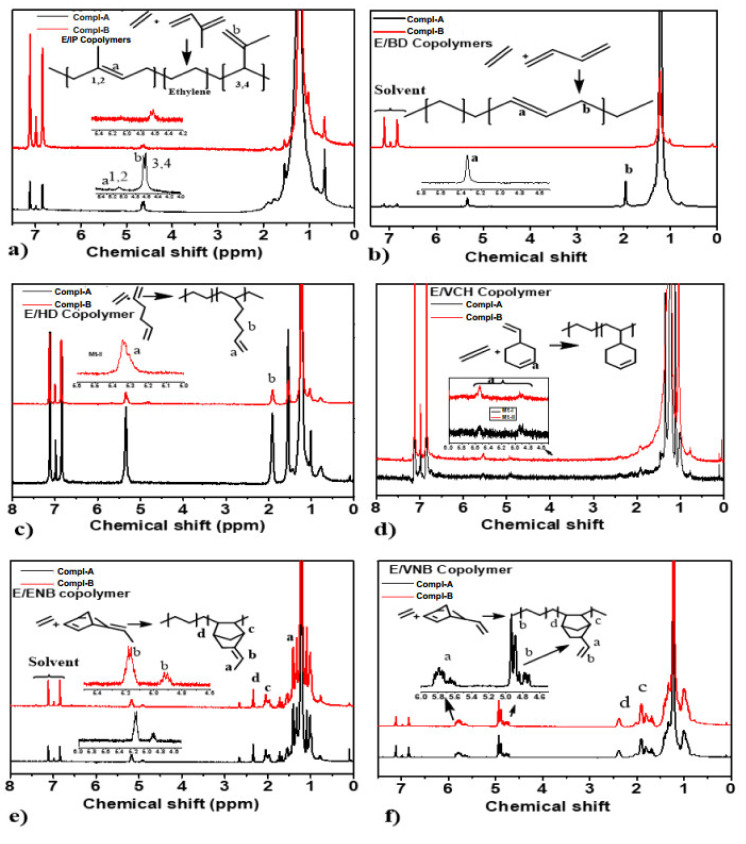
NMR spectra and chain structure of E/diene copolymers.

The ^1^H NMR spectroscopy technique was used to elucidate the chain structure of the polymer. As shown in Figure 4, ^1^H NMR indicates the effective incorporation of dienes in the polymer backbone. The polyethylene protons appear at 1.01–1.30 ppm, while the signals between 5.6 and 4.4 ppm are assigned to dienes (conjugated and nonconjugated); see Figure 4. These proton signals are absent in polyethylene or ethylene/propylene copolymerizations. The peaks of trans-1, 4-isoprene units are much higher than those of the 3, 4-isoprene units indicating the regioselectivity of conjugated (isoprene) insertion; see Figure 4a. However, the 1, 4-isoprene units’ content gradually increases with an excess of isoprene concentration in the system. Furthermore, 3, 4-isoprene units gradually decreased with increasing isoprene concentration. In the case of a nonconjugated diene, double bonds in HD could chelate with a metal center. Chelation is difficult for VCH and ENB due to the low steric flexibility of the endo double ring. Moreover, cyclic nonconjugated dienes (ENB, VNB) exhibiting a strained double bond are reactive in olefin polymerizations and ideally incorporate in the polymer backbone at 5.2 to 5.4 ppm. However, unreactive double bonds (1, 2 disubstituted or trisubstituted) of nonconjugated dienes can be utilized for different postpolymerization techniques.

The molecular weight (Mw) of the E/IP copolymer synthesized with complex A is higher than that of the nonconjugated diene. However, nonconjugated diene effects are much more important for complex B, e.g., the E/ENB copolymer produced higher Mw, while E/VNB obtained a low Mw. Overall, the molecular weight of the EP copolymer was higher with complex A than with complex B; see Table 1 and Table 2. According to previous studies, the E/diene copolymer synthesized with complex B/MMAO has lower Mw when using the same catalyst. Complex A produced E/conjugated diene and E/nonconjugated diene copolymers with higher Mw than complex B due to the presence of electron-donating substituents in the ligand and the stronger steric hindrance of complex A, which helps depress the chain transfer reactions of the active centers. Furthermore, in this system, the Mw of E/diene copolymers decreased with the increase in diene bulkiness, meaning that a chain transfer reaction with nonconjugated dienes should be faster than with conjugated dienes. However, the larger steric bulkiness in ENB, VNB, and VCH could be the main reason for their different chain transfer efficiencies. 

**Figure 5:**
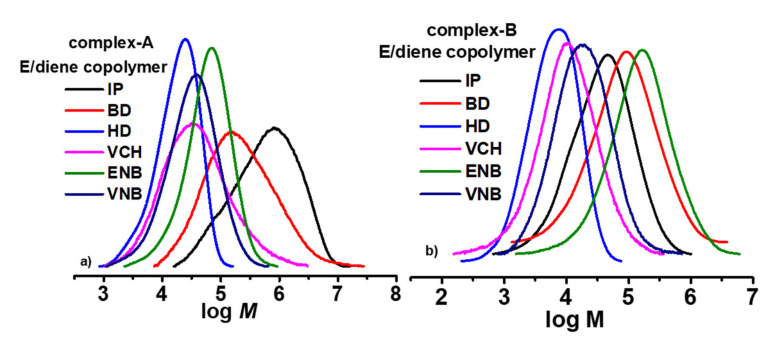
Molecular weight distribution (MWD) for ethylene/diene (conjugated and nonconjugated) terpolymers produced with (**a**) complex A and (**b**) complex B.

The different steric bulkiness can explain the chain transfer reactions with nonconjugated dienes. With the lower steric hindrance of VNB and ENB at endo bond, the active site’s surroundings became sterically less crowded, and the rate of chain transfer became faster, resulting in E/nonconjugated diene copolymers with lower Mw. Complex A, for both types of diene (conjugated or nonconjugated), had more considerable steric hindrance, so the chain transfer reaction became slower, leading to copolymers with higher Mw. However, both double bonds in HD could chelate with the metal center, resulting in both catalysts producing lower Mw in the copolymer. A smaller steric hindrance in BD would be more favorable for fast chain transfer reaction than IP; IP with a methyl group has a greater steric hindrance, leading to higher molecular weight than BD. Moreover, the molecular weight distributions (MWD) of E/conjugated copolymers range from 3 to 4, while for E/nonconjugated copolymers it is 2‒3; both types of copolymers are monomodal (see Figure 5), indicating a single-site metallocene catalyst and an obtained product that is copolymer in nature.

### 3.3. Ethylene, Propylene and Diene Monomer Terpolymerization

Ethylene‒propylene and diene (conjugated and nonconjugated) terpolymerization was carried out under similar conditions, as shown in Table 3.

**Figure 6:**
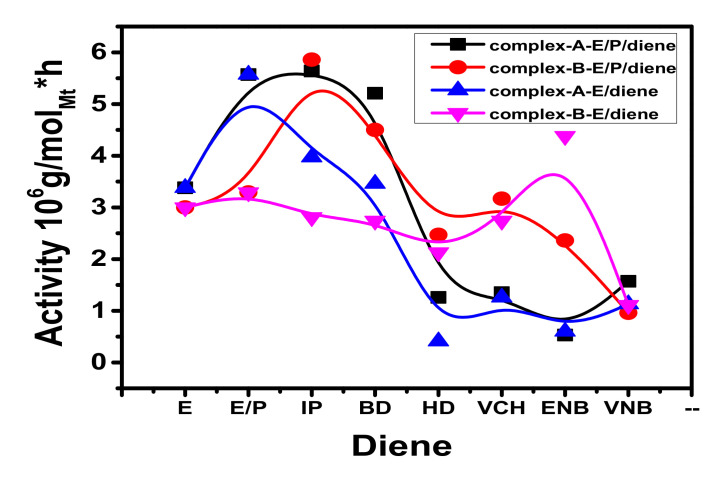
The activity of ethylene/propylene diene (conjugated and nonconjugated) terpolymerization with complex A and complex B at ethylene 80/20 mol% and 0.06 mol/L diene.

The effects of conjugated and nonconjugated dienes on ethylene-propylene copolymerization behavior were investigated at a constant feed mole ratio (E/P 80/20) as shown in Table 3. The catalytic activity improved to an optimal value of 5.64 × 10^6^ g/mmol_Mt_·h with IP. It was found that the addition of IP and ENB at 0.06 mol/L resulted in slightly increased polymeric catalytic activity, as demonstrated in Figure 6. The maximum polymerization activity was obtained with 0.03 to 0.06 diene mol/L; beyond this value, the polymerization activity was reduced, as investigated by Ahmadjo et al. and Fan et al. [43]. In general, when numerous types of dienes were added as a third monomer, the activity was dramatically reduced compared to the conventional EP [45,46]. Propylene addition resulted in slightly increased polymeric catalytic activity. According to our previous study, the increase in polymerization was more prominent when increasing the ratio of propylene, possibly due to ethylene’s lower reactivity in the presence of dienes. The activity of ethylene and propylene homopolymerization was lower than ethylene‒propylene copolymerization due to the comonomer effect, as investigated by Waymouth and Kravchenko et al. [30,34].

**Figure 7:**
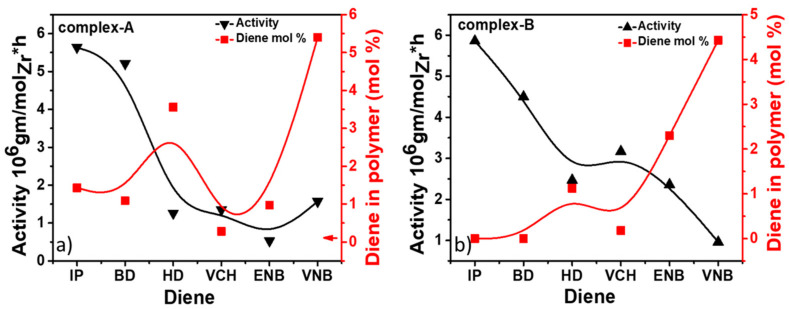
Variation of activity profile and diene content in ethylene/propylene/diene with (**a**) complex A and (**b**) complex B.

The conjugated dienes have achieved the highest polymerization activities compared to the nonconjugated dienes in the presence of complex A. However, IP produced the most increased activity among the dienes, while ENB presented the most substantial deactivation effect (see Figure 7). Jongsomjit et al. suggested that the addition of VCH led to the highest polymerization yield among the other dienes, whereas HD led to the lowest activity for complex B/MAO [34] similar results were reported by Fan et al. for E/P/ENB complex B/MMAO [43]. They found that the presence of the nonconjugated diene ENB considerably curtails the catalytic activity by 5–10-fold compared to E/P copolymers. The activity order of nonconjugated dienes was VCH > VNB > HD > ENB for complex A/borate/TIBA. On the other hand, for complex B/borate/TIBA it was ENB > VCH > HD > VNB. The nonconjugated polymerization activities in the presence of VCH lay in between those of ENB and VNB. The order suggests that both double bonds in HD could be chelated, with a metal center. Chelation is difficult for VCH, ENB, and VNB due to the low steric flexibility of the endo double ring’s bond. The extent of activation caused by IP and BD was stronger in complex A than in complex B. However, the degree of deactivation initiated by ENB, VNB, VCH, and HD was also greater in complex A than in complex B. Overall, the catalytic activity of complex A was more sensitive to nonconjugated dienes than to conjugated dienes (see Figure 7).

The effects of conjugated IP and BD and nonconjugated ENB, VNB, VCH and HD dienes on both catalysts were sharply different. However, IP and BD showed the weakest deactivation effect on both catalysts. Still, the insertion rate of IP and BD was low when complex B was the catalyst (see Figure 7), which might be due to the lower steric hindrance of complex B chelation of both double bonds with metal atoms through conjugation phenomena. It was noted that EPDM technically required amounts of 5–10 mol%. In addition, complex A produced polymerization of E/P/IP with higher conversion of synthesizing polymers with a 3, 4 IP unit microstructure and a low number of 1, 4 IP units. In contrast, under similar polymerization conditions, complex B is significantly less able to insert the conjugated diene in the backbone of polymer chains. Still, the polymerization activity is comparable to that of complex A (see Figure 7). Notably, both metallocenes show suitable catalytic activities in the co- and terpolymerization and present a comonomer effect. This was mainly analyzed in the co/terpolymerization with complex A. However, complex B showed no dramatic drop in polymerization activity when the dienes concentration in the feed was increased.

It is important to relate the activities of ethylene‒propylene nonconjugated terpolymerization catalyzed with complex B/TIBA/borate and complex B/MAO as well as complex B/MMAO. The activity of nonconjugated dienes was ENB: 1.9 × 10^6^, VCH: 2.2 × 10^6^, and HD: 1.8 × 10^6^ g/mmol_Mt_·h (E/P feed ratio: 74/26 in toluene, reaction temperature: 50 °C, time: 1 h). Our group previously reported on E/P/ENB terpolymerization with a complex B/MMAO system; the catalytic activity was 1.0 × 10^6^ g/mmol_Mt_·h (E/P: 70/30; pressure: 5 bar, [ENB] 0.03 mol/L n-heptane as the solvent), which is close to the activity of complex B/TIBA/borate. It is recommended that borate and aluminoxane are effective catalysts for ethylene/propylene/nonconjugated terpolymerizations.

### 3.4. Chain Structure of Terpolymers

**Figure 8:**
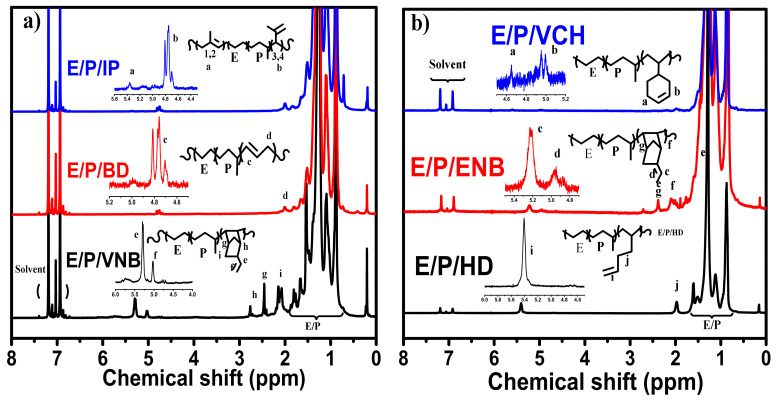
Chain structure of ethylene/propylene/diene terpolymerizations.

**Figure 9:**
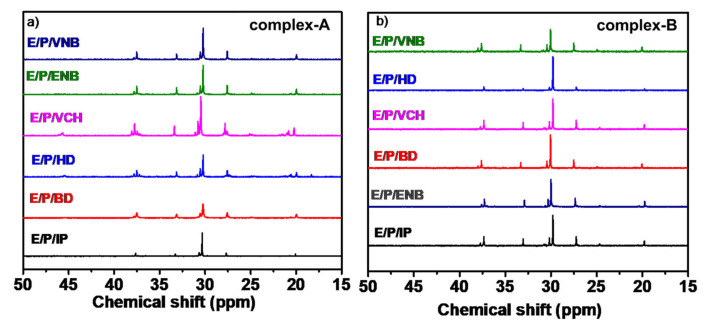
^13^C-NMR spectrum for ethylene/propylene/diene (conjugated and nonconjugated) terpolymers produce with (**a**) complex A and (**b**) complex B.

The co-/terpolymers were analyzed with ^1^H NMR and ^13^C NMR techniques to investigate the chain structure and their correlation with the metallocene structure. Figure 8 demonstrates the ^1^H NMR spectrum of ethylene/diene copolymers produced at a dienes concentration of 0.06 mol/L. The ^13^C NMR spectrum of ethylene/propylene/diene terpolymerization produced at an ethylene/propylene ratio of 80/20 with a dienes concentration of 0.06 mol/L under similar conditions is shown in Figure 9.

As we observed, the E/P/diene terpolymers have a diene content no greater than that of ethylene/diene copolymers (see Table 1, Table 2 and Table 3), while ^13^C NMR signals from the assimilated diene units as well as sequences containing diene units could not be detected. Moreover, only the comonomer sequences exhibiting E and P units are present in their spectrum. However, the comonomer sequence distributions of ethylene/propylene/diene (conjugated and nonconjugated) terpolymer were determined depending on the ^13^C NMR peak intensity [27,43]. Based on our data, the effects of conjugated and nonconjugated dienes may be assumed from the subsequent polymerization behavior and ethylene‒propylene incorporation. It was observed that adding a small amount of conjugated and nonconjugated dienes to a metallocene/borate/TIBA catalyst system could alter the terpolymers’ behavior. It should be noted that the effects of diene addition were based on the types of dienes and the E/P ratios. However, we agree that a more effective system such as steady-state isotopic transient kinetic analysis (SSITKA) will be beneficial to gain a deeper understanding of the process (Shannon and Goodwin, 1995; Belambe et al., 1997; Jongsomjit et al., 2003), to make it possible to define the reaction intermediates [34]. However, additional instrumentation, e.g., a mass spectrometer along with the isotope (^13^C) of the corresponding reactants, is needed for this technique. Therefore, this instrument and device can be used to perform further studies.

The E/P/diene triad sequence distributions, reactivity ratios, and average sequence length obtained from the sequence distribution are summarized in Table 4.

We can observe that the different dienes as termonomers initiated the evident decline of PPP triads and rise of EEE triads; meanwhile, the average length of (PP) sequences (*n*_P_) decreased. When complex A was polymerized, the E/P/diene caused a significant decline in the (*r*_E_ × *r*_P_), meaning that the trend of ethylene block copolymerization was reversed by the diene (conjugated and nonconjugated), and the diene may change the mechanism of ethylene/propylene copolymerization. On the other hand, when complex B was the catalyst, the addition of dienes such as VCH and ENB did not affect the reactivity ratios (*r*_E_ × *r*_P_). This fact can be considered confirmation that the addition of VCH and ENB as termonomers in the propagation polymer chain had only minor effects on ethylene/propylene copolymerizations. However, HD brings about a significant decrease in *r*_E_ × *r*_P_. We see that nonconjugated dienes VCH and VNB had a lower deactivation influence on ethylene/propylene copolymerizations than HD in complex B.

Similarly, in complex B, *r*_E_×*r*_P_ decreased with BD and IP in the system, and the average polypropylene sequence length decreased to <1.0. (see Table 4). Finally, the addition of diene in the E/P/diene terpolymerizations led to a limitation on propylene insertion and coordination in both catalysts. The reactivity ratio values of ethylene (*rE*) with complex A were not much affected, while *rE* was reduced with nonconjugated diene for complex B (see Figure 9b). This means that the nonconjugated dienes had more influence on the incorporation of ethylene than conjugated dienes.

**Figure 10:**
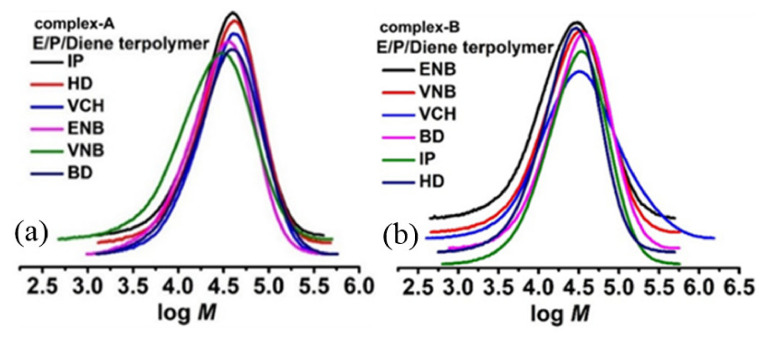
Molecular weight distribution (MWD) for ethylene/propylene/diene (conjugated and nonconjugated) terpolymers produced with (**a**) complex A and (**b**) complex B.

The molecular weight of the E/diene copolymers is higher than that of the E/P/diene terpolymers. Mw of EPDM is one of the critical structural parameters defining its physical properties. Moreover, sufficient Mw is required for the EPDM to work as an elastomer. As seen in Table 1, the E/conjugated diene (E/IP) copolymer produced with complex A has higher Mw than that of nonconjugated dienes. On the other hand, the E/ENB synthesis EMDM with both catalysts showed higher Mw than ENB, VCH, and HD. In the case of ethylene/propylene/diene terpolymerization, the terpolymers produce with complex A in the presence of IP showed higher molecular weight; however, complex B, interestingly, presented higher Mw when ENB and VCH were termonomers. According to the literature, the Mw level of the EPDM was significantly related to the co/terpolymers’ composition. According to our previous kinetic study, the co/terpolymers with higher E content presented higher Mw since the chain transfer reaction is much faster with propylene than with ethylene [4]. In the EPDM with ENB, HD, and VCH injected as termonomers with complex B, the Mw was much higher than for the other terpolymers due to the higher ethylene content (Figure 10). These Mw results agree with those previously reported for complex B/MAO [34].

**Figure 11:**
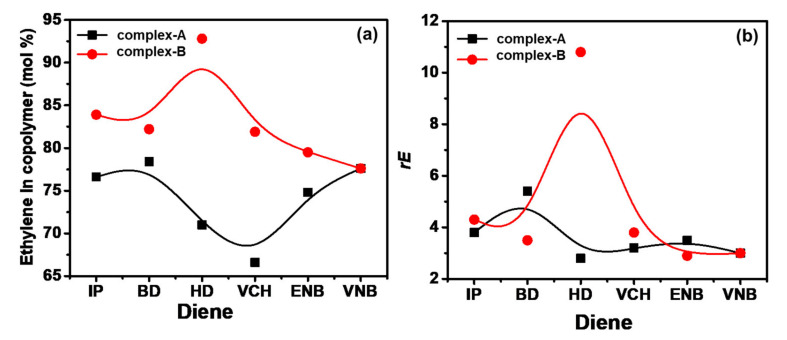
(**a**) Ethylene content in terpolymer produced with conjugated and nonconjugated dienes; (**b**) change in reactivity ratio rE in terpolymers with the addition of dienes.

Although the MWD of EPDM generated by complex B was narrow compared to complex A, its polydispersity index (Ð) value was still larger than 2, the theoretical (Ð) of a true single-site metallocene catalyst, suggesting the existence of several active sites in the catalytic framework (see Figure 11). 

As compared with the Mw of EPDM synthesized through complex B/MMAO under identical conditions, the complex B/TIBA/borate catalyst system showed EPDM with markedly lower Mw but similar MWD [7,34,43].

## 4. Conclusions

In conclusion, the copolymerization of ethylene/diene (conjugated and nonconjugated) and ethylene/propylene/diene terpolymerization were successfully symmetrically performed by the complexes, namely, rac-Me_2_Si (2-Me-4-Ph-Ind)_2_ZrCl_2_- (complex A) and rac-Et(Ind)_2_ZrCl_2_- (complex B), in combination with 2 equiv. of borate. The addition of a small amount of conjugated and/or nonconjugated dienes might change the behaviors of co- and terpolymers in a metallocene/TIBA/borate catalyst system. In complex A catalyzed by co- and terpolymerization, HD, IP and VNB are much more reactive than other dienes and incorporated more quickly in the polymer backbone. However, ENB exerted the most substantial deactivation effect on complex A, and VNB caused the strongest deactivation in complex B. However, the high sensitivity of complex A toward nonconjugated dienes significantly limits complex A utilization with higher ENB concentrations. For the first time, we have also shown that conjugated dienes (IP and BD), mainly used in the manufacturing of EPDM, can significantly increase complex A utilization. The maximum yield of both catalysts was obtained with conjugated dienes IP and BD, and 1, 2 insertions of IP are higher than those of 1, 4, which are usually not available in coordination polymerization. E/P/diene terpolymers with high propylene contents of 20–25 mol% and varying conjugated and nonconjugated dienes at 2–10 mol% were achieved in the presence of complex A/TIBA/borate. Similarly, using complex B with a less steric ligand structure, E/P/diene terpolymers with comparatively lower propylene contents of 17–21 mol% and nonconjugated diene contents of 1–4 mol% were achieved. The highest catalytic activity of 5.38‒5.86 × 10 g/mol_MT_ * h was obtained in the terpolymerization of E/P/conjugated for both catalysts rather than E/P/nonconjugated and copolymerization of ethylene diene. Complex A catalyzed E/IP copolymerization showed the highest molecular weight, 952,946 g/mol, while complex B catalyzed with E/BD gave the lowest molecular weight, 5360 g/mol. Efficiency in terms of co- and termonomer incorporation with higher molecular weight was found for complex A.

## Figures and Tables

**Table 1:** Ethylene-diene copolymerizations catalyzed with *rac*-Me_2_Si(2-Me-4-Ph-Ind)_2_ZrCl_2_ (complex A)/Ph_3_C][B(C_6_F_5_)_4_/TIBA ^a^).

Run	Diene Type	Diene (mol/L)	Yield (g)	Activity (10^6^ g/mmol_Mt_·h)	Diene ^b^ (mol%)	*M*_w_^c^ (kg/mol)	Ɖ ^c^
1.1	E	0	1.41	3.38	0.0	244.4	8.7
1.2	IP	0.06	1.61	3.97	N/A	N/A	N/A
1.3	IP	0.12	1.35	3.27	1.08	952946	3.98
1.4	BD	0.06	1.43	3.46	N/A	N/A	N/A
1.5	BD	0.12	1.11	2.69	0.60	445359	4.65
1.6	HD	0.06	0.17	0.41	N/A	N/A	N/A
1.7	HD	0.12	0.11	0.26	14.81	23586	1.99
1.8	VCH	0.06	0.52	1.26	N/A	N/A	N/A
1.9	VCH	0.12	0.23	0.55	1.55	36479	3.1
1.10	ENB	0.06	0.25	0.60	1.12	85226	2.21
1.11	ENB	0.12	N/A	N/A	N/A	N/A	N/A
1.12	VNB	0.06	0.47	1.13	N/A	N/A	N/A
1.13	VNB	0.12	0.19	0.46	2.70	48676	2.59

^a^ Reaction conditions: Metallocene complex A = 1.25 μmol, Borate activator = 2.5 μmol, ethylene pressure = 0.1 MPa, polymerization reaction time *t*_p_ = 20 min, solvent toluene = 50 mL, co-catalyst TIBA 1000 μmol, ^b^ Determined by high-temperature ^1^HNMR, ^c^ Determined by high-temperature GPC, N/A = Not applicable.

**Table 2:** Ethylene-diene copolymerizations with *rac*-Et(Ind)_2_ZrCl_2_(complex B)/Ph_3_C][B(C_6_F_5_)_4_/TIBA ^a^.

Run	Diene Type	Diene (mol/L)	Yield (g)	Activity (10^6^gm/mmol_Mt_·h)	Diene ^b^ (mol%)	M_w_^c^ (kg/mol)	Ɖ^c^
2.1	E	N/A	1.26	3.00	N/A	88	4.4
2.2	IP	0.06	1.17	2.81	N/A	N/A	N/A
2.3	IP	0.12	1.16	2.78	0.41	65	3.52
2.4	BD	0.06	1.13	2.74	N/A	N/A	N/A
2.5	BD	0.12	1.10	2.26	00	80	4.12
2.6	HD	0.06	1.05	2.13	N/A	N/A	N/A
2.7	HD	0.12	0.29	0.70	3.4	5	2.08
2.8	VCH	0.06	1.14	2.74	N/A	N/A	N/A
2.9	VCH	0.12	0.42	1.01	0.86	16	3.06
2.10	ENB	0.06	1.81	4.38	N/A	N/A	N/A
2.11	ENB	0.12	1.53	3.70	3.45	84	3.87
2.12	VNB	0.06	0.46	1.11	N/A	N/A	N/A
2.13	VNB	0.12	0.36	0.87	4.65	10	2.31

^a^ Reaction conditions: Metallocene catalyst complex B = 1.25 *μ*mol, Borate activator = 2.5 μmol, ethylene pressure = 0.1 MPa, polymerization reaction time *t*_p_ = 20 min, solvent toluene = 50 mL, co-catalyst TIBA 1000 μmol, ^b^ Determined by high-temperature ^1^HNMR, ^c^ Determined by high-temperature GPC. N/A = Not applicable.

**Table 3:** Ethylene, propylene and diene (conjugated and nonconjugated) terpolymerization ^a^.

Run	Complex	Diene Type	Yield (g)	Activity (10^6^ g/mmol_Mt_·h)	Diene ^b^ (mol%)	E mol%	*M*_w_ (kg/mol)	Ɖ
3.1	A	E/P	2.32	5.57	0	77.9	18.1	3.3
3.2	A	IP	2.33	5.64	1.43	76.6	94.2	2.49
3.3	A	BD	2.15	5.21	1.09	78.4	50.8	3.40
3.4	A	HD	0.52	1.26	3.56	71.0	41.6	2.08
3.5	A	VCH	0.56	1.35	0.28	66.6	49.1	2.15
3.6	A	ENB	0.22	0.53	0.97	74.8	38.1	2.09
3.7	A	VNB	0.65	1.57	5.4	77.6	25.3	2.59
3.8	B	E/P	1.37	3.29	N/A	79.0	13.9	2.5
3.9	B	IP	1.34	3.22	N/A	83.9	39.8	2.38
3.10	B	BD	1.27	3.05	N/A	82.2	N/A	N/A
3.11	B	HD	1.02	2.47	1.12	92.2	41.3	2.11
3.12	B	VCH	1.31	3.14	0.18	81.9	51.0	2.79
3.13	B	ENB	1.48	3.55	2.30	79.5	35.3	2.52
3.14	B	VNB	0.40	0.96	4.43	77.6	23.5	3.50

^a^ Reaction conditions: metallocene catalysts (complexes A and B) = 1.25 μmol, Borate activator = 2.5 μmol, ethylene/propylene pressure = 0.1 MPa, ethylene/propylene mole ratio 80/20, polymerization reaction time *t*_p_ = 20 min, solvent toluene = 50 mL, co-catalyst TIBA 1000 μmol, ^b^ Determined by high-temperature ^1^HNMR, ^c^ Determined by high-temperature GPC. N/A = Not applicable.

**Table 4:** Sequence distribution of terpolymers determined by ^13^C NMR.

Run	Diene	Complex	EEE	PEE+EEP	PEP	EPE	EPP+PPE	PPP	E	P	re	rp	rerp	nP ^a^	nE ^a^
3.1	IP	A	45.2	24.9	6.5	14.6	2.8	1.2	76.6	18.6	3.8	0.2	0.6	4.1	1.0
3.2	BD	A	47.2	24.1	7.1	17.5	2.3	1.8	78.4	21.6	5.4	0.1	0.5	4.2	1.2
3.3	HD	A	35.6	28.7	6.7	12.6	16.2	0.2	71.0	29.0	2.8	0.4	1.0	3.5	1.4
3.4	VCH	A	31.7	25.4	6.5	12.7	15.4	5.3	66.6	34.4	3.2	0.6	1.8	3.6	1.8
3.5	ENB	A	42.2	23.1	9.5	18.5	3.2	1.4	74.8	23.2	3.5	0.1	0.3	3.8	1.2
3.6	VNB	A	44.6	25.4	7.6	19.4	2.0	1.0	77.6	22.4	3.0	0.1	0.3	3.9	1.1
3.7	IP	B	58.8	19.6	5.5	15.4	0.4	0.4	83.9	16.1	4.3	0.03	0.1	5.3	1.0
3.8	BD	B	54.0	23.4	4.8	16.4	0.5	0.9	82.2	17.8	3.5	0.1	0.2	4.7	1.0
3.9	HD	B	80.1	12.5	0.2	6.5	0.2	0.6	92.8	7.2	10.8	0.1	1.2	12.9	1.0
3.10	VCH	B	52.9	24.5	4.5	16.6	0.7	0.8	81.9	18.1	3.8	0.1	0.2	4.7	1.0
3.11	ENB	B	48.5	23.7	7.3	18.3	1.7	0.4	79.5	20.5	2.9	0.1	0.2	4.0	1.0
3.12	VNB	B	44.6	25.4	7.6	19.4	1.9	1.1	77.6	22.4	3.0	0.1	0.3	3.9	1.1

^a^ Average length of polyethylene and polypropylene sequences.

## Data Availability

Data will be made available on request.
